# Assay Optimization Can Equalize the Sensitivity of Real-Time PCR with ddPCR for Detection of *Helicoverpa armigera* (Lepidoptera: Noctuidae) in Bulk Samples

**DOI:** 10.3390/insects12100885

**Published:** 2021-09-29

**Authors:** Thayssa M. R. Oliveira, Frida A. Zink, Renato C. Menezes, Érico C. Dianese, Karina C. Albernaz-Godinho, Marcos G. Cunha, Alicia E. Timm, Todd M. Gilligan, Luke R. Tembrock

**Affiliations:** 1 Escola de Agronomia, Universidade Federal de Goiás, Goiânia 74690-900, Brazil; thayssa28@hotmail.com (T.M.R.O.); renato_cmenezes@hotmail.com (R.C.M.); erico.dianese@gmail.com (É.C.D.); kcalbernaz@gmail.com (K.C.A.-G.); mgcagro@gmail.com (M.G.C.); 2 Department of Agricultural Biology, Colorado State University, Fort Collins, CO 80523, USA; frida.zink@colostate.edu (F.A.Z.); alicia.timm@colostate.edu (A.E.T.); 3 USDA-APHIS-PPQ-Science & Technology, Identification Technology Program, Fort Collins, CO 80526, USA

**Keywords:** invasive species, agriculture, rDNA, *Helicoverpa zea*, Old World bollworm

## Abstract

**Simple Summary:**

Invasive species are a constant threat to agriculture throughout the world against which early detection is one of the primary defenses. The Old World bollworm is one of the most important invasive agricultural pests in the world. While historically absent from the Americas, this species was first found in South America in 2013 and poses an ongoing threat of spreading into North America. Surveys are conducted each year, which result in hundreds or thousands of traps that must be screened for this species. Unfortunately, the most common non-target is the native corn earworm, which is nearly identical morphologically to the Old World bollworm and cannot be easily separated. Molecular methods have been developed to screen these trap samples, but the required equipment is expensive and not commonly available. This study details improvements to current molecular methods that will allow for screening of bulk trap samples using standard laboratory instruments and protocols. The ability to perform these methods in nearly any molecular biology lab will greatly enhance our ability to detect and exclude this important pest.

**Abstract:**

*Helicoverpa armigera* (Hübner) is one of the most important agricultural pests in the world. This historically Old World species was first reported in Brazil in 2013 and has since spread throughout much of South America and into the Caribbean. Throughout North America, *H. armigera* surveys are ongoing to detect any incursions. Each trap is capable of capturing hundreds of native *Helicoverpa zea* (Boddie). The two species cannot be separated without genitalic dissection or molecular methods. A ddPCR assay is currently used to screen large trap samples, but this equipment is relatively uncommon and expensive. Here, we optimized a newly designed assay for accurate and repeatable detection of *H. armigera* in bulk samples across both ddPCR and less costly, and more common, real-time PCR methods. Improvements over previously designed assays were sought through multiple means. Our results suggest bulk real-time PCR assays can be improved through changes in DNA extraction and purification, so that real-time PCR can be substituted for ddPCR in screening projects. While ddPCR remains a more sensitive method for detection of *H. armigera* in bulk samples, the improvements in assay design, DNA extraction, and purification presented here also enhance assay performance over previous protocols.

## 1. Introduction

Corn earworm, *Helicoverpa zea* (Boddie) (Lepidoptera: Noctuidae), is considered one of the most damaging insect pests in North America. The species is highly polyphagous and has been recorded feeding on more than 120 species of plants, including many economically important crops such as maize, sorghum, tomato, and cotton [1,2,3,4]. *Helicoverpa zea* is restricted to the Western hemisphere and was the only major *Helicoverpa* pest species present in this region until 2013, when Old World bollworm, *H. armigera* (Hübner), was recorded in Brazil [5,6]. *Helicoverpa armigera* is considered native to Europe and Asia and is also present throughout Africa, Australia, and Oceania [7]. Larvae of this species have an even broader host range than those of *H. zea*, with over 180 species of host plants recorded, including many specialty crops [4]. This broad host range has allowed *H. armigera* to spread rapidly throughout much of South America and the Caribbean [8]. The first U.S. interception was recorded in Puerto Rico [9] raising concern that the species would soon be detected within the continental U.S. This concern was realized in 2015 when three individuals of *H. armigera* were recorded in Florida [10]. Subsequent surveys in Florida have shown, however, that the species has so far failed to become established. Nevertheless, it is likely that *H. armigera* will continue to increase its geographic range in the New World given that adults are highly mobile and capable of flying 20–40 km in one day [11]. Human-mediated introductions from the movement of passengers and trade goods may also result in *H. armigera* increasing its geographic range [12,13] as the species is often intercepted at U.S. ports of entry [14]. Based on habitat and host suitability, *H. armigera* could become established in many states in the continental U.S. [15], where it would substantially threaten important agricultural crops [16,17]. Preventing this species from increasing its geographic range and further establishing itself in the Western hemisphere is of utmost importance.

The most important factors for initiating a rapid response to an incursion of *H. armigera* are prompt detection and ongoing screening. *Helicoverpa armigera* and *H. zea* are nearly identical morphologically, which makes morphological identification impossible without genitalic dissections. The time constraints and technical expertise required for identification by genitalic dissection [7,18,19] can result in *H. armigera* remaining undetected in areas where *H. zea* is present (i.e., most of the Western hemisphere). Molecular methods are therefore increasingly used to distinguish between *H. zea* and *H. armigera*.

As the range of *H. armigera* expands across South America, the Caribbean, and likely into North America [6,13,16,20], it is essential to have multiple means of screening many individuals in a short period of time. Such screening efforts provide useful data for targeted responses to the spread of *H. armigera* as well as detailed information in understanding the range expansion dynamics for this economically important pest. The use of ddPCR (droplet digital PCR) to detect very small amounts of target DNA among large quantities of non-target DNA has been demonstrated in numerous applications (e.g., [21,22,23]) including the detection of *H. armigera* in bulk samples [24]. While the use of ddPCR is an attractive method for pest detection in bulk samples due to its accuracy, tolerance to PCR inhibitors, and method of direct standard-free quantification [25], it is costly, and the systems are not widely available. Conversely, real-time PCR, because of the relatively low cost of machine ownership and operation, is frequently utilized for detection of pest species of many types (e.g., [26,27,28]). Unfortunately, the real-time PCR-based methods for detecting *H. armigera* in bulk samples do not perform well in real-world sized samples which routinely include hundreds of specimens [24,29]. As such, an accurate DNA-based identification method that can be employed across either ddPCR or real-time PCR is needed to provide flexibility and compatibility across labs and instruments in screening bulk samples for detection of *H. armigera*.

Given the need for a more flexible and sensitive method to screen bulk samples, we sought to optimize a newly designed assay for accurate and repeatable detection of *H. armigera* in bulk samples to more closely match the efficiency of real-time PCR to ddPCR. Improvements over previously designed assays were sought through changes to primer and probe design, DNA extraction method, and assay output interpretation. Effects of different treatments were measured through increases in assay sensitivity and compared within each assay, across assays, and to previously developed assays where applicable. Since the current standard is to screen bulk samples via ddPCR followed by real-time PCR of individuals in bulk samples positive for *H. armigera*, the process is bottle necked by the lack of ddPCR systems available to identifiers. An optimized real-time PCR assay for use with bulk samples, as presented here, could vastly improve early detection of an invasion of *H. armigera*.

## 2. Materials and Methods

### 2.1. Origin and Type of Insect Material

The DNA for this study was extracted from the legs of adult specimens of *H. armigera* and *H. zea*. *Helicoverpa zea* specimens were collected in the summer of 2016 in Colorado and Minnesota and in the autumn of 2019 in Florida. Samples were collected using UNI-Trap Multi-Color (Alpha Scents, Inc., Canby, OR) bucket traps baited with *H. armigera* pheromone lure formulated and produced by the USDA Forest Pest Methods Laboratory in Buzzards Bay, MA. After collection, samples were stored dry in paper envelopes at 4 °C prior to use. All specimens were identified to genus visually and confirmed to be *H. zea* by ddPCR using the method outlined by Zink et al. [24]. Specimens of *H. armigera* were acquired from lab colonies at the USDA-APHIS Forest Pest Methods Laboratory, Cape Cod, Massachusetts, established from Spanish specimens, and the Max Planck Institute of Chemical Ecology in Jena, Germany, established using *H. armigera* from Queensland, Australia. Additional *H. armigera* were collected from various locations throughout South Africa (Supplementary Table S1). After collection, *H. armigera* samples were preserved in 100% ethanol in individual microcentrifuge tubes and stored at −80 °C until use.

### 2.2. DNA Extraction from Individual Specimens

For the samples used in optimization steps, DNA was extracted from two legs of individual specimens of *H. armigera* or *H. zea* using the Qiagen DNeasy Blood & Tissue Kit (Qiagen, Valencia, California) following manufacturer’s instructions. DNA concentrations were determined using a Nanodrop 2000 (Thermo Fisher Scientific, Waltham, MA, USA). The samples used for testing assay sensitivity were extracted using four legs from individual specimens of *H. armigera* using the Lucigen MasterPure Complete DNA and RNA Purification Kit (LGC Biosearch Technologies, Novato, CA, USA) following manufacturer’s instructions with an additional 1-h incubation at −20 °C before DNA precipitation to increase total DNA yield. DNA concentrations were determined using a Qubit Fluorometer with the dsDNA HS Assay Kit (Thermo Fisher Scientific) following manufacturer’s instructions. DNA was stored at −20 °C until use and then archived at −80 °C.

### 2.3. Bulk DNA Extraction

To identify the best bulk DNA isolation method for species-specific real-time PCR detection, we evaluated adjustments to the formulation and method of squish buffer DNA extraction [30]. The original squish buffer concentrations of 10 mM Tris-HCl, 1 mM EDTA, 25 mM NaCl, and pH 8.2 as well as the modified formulation of Perera et al. [29], wherein EDTA and NaCl concentrations reduced by 50%, were tested. In addition, we compared EDTA and NaCl concentrations of 2×, 5×, and 10× the concentration of the original Gloor et al. [30] formulation. In all tests, Proteinase K was excluded which differs from Gloor et al. [30].

Samples were prepared by adding 1 leg of *H. armigera* to increasing numbers of *H. zea* legs along with several 2.3 mm zirconia/silica beads in 1.5 mL microcentrifuge tubes. The samples were pulverized for 2 min on high speed in a mini-beadbeater (Biospec Products, Bartlesville, OK, USA). After grinding to a fine powder, 10 µL/leg of squish buffer was added to the tube, and it was incubated overnight at 80 °C (56 °C equivalent) and shaking at 500 rpm in a dry bath Thermomixer FP (Eppendorf AG, Hamburg, Germany). After incubation, the samples were spun down at 2152, 8609, or 16,873× *g*, for 10 min to pellet debris in an Eppendorf 5418 bench-top microcentrifuge (Eppendorf AG).

The best performing method, a modified squish buffer with 125 mM NaCl, 5 mM EDTA, and 10 mM Tris-HCl with a centrifugation step at 8609× *g* for ten minutes, was repeated for eight ratio extractions (*H. armigera*: *H. zea*) from 1:0 to 1:500.

A set of 59 samples containing one leg of *H. armigera* and 50 legs of *H. zea* were extracted using the best-performing modified squish buffer method. The *H. armigera* legs used were collected from disparate geographic locations as described above and were of varying quality to account for DNA degradation that occurs in trap samples (Supplementary Table S1). Because the squish buffer DNA extractions lack the purification that takes place in a column extraction, a 100 µL aliquot from each of these extractions was further purified using AMPure XP paramagnetic beads following the manufacturer’s workflow (Beckman Coulter, Danaher Corporation, Brea, CA, USA).

### 2.4. Primer and Probe Design

A new set of primers and probes was designed in the same region as other successful molecular assays which had used a portion of ITS1 and the 5′-flanking 18S rDNA region to differentiate *H. armigera* from sister species *H. zea* and relatives [29,31]. Alignments of sequences from *H. armigera* and related species (*H. zea*, *H. assulta*, *Chloridea subflexa,* and *C. virescens*), as well as intra-genome tandem rDNA repeats between *H. zea* and *H. armigera* from whole genome alignments (conducted *post hoc*), were utilized to find and later confirm consistent differences between samples. Primers and probes were designed using Geneious v8.1.9 (https://www.geneious.com). The program Primer 3 v2.3.7 [32] was used to calculate Tm with the SantaLucia [33] method, and OligoCalc [34] was employed to test for self-annealing and hairpin formation. Any primers and probes that were found to have poor structural qualities (e.g., many self-annealing sites) were not tested or were repositioned over the variable sites to exclude predicted structural faults. Primers and probes were synthesized by IDT (Integrated DNA Technologies, Coralville, IA, USA). Initial real-time PCR experiments were performed with 2 µL of DNA extracted from *H. armigera* only, *H. zea* only, and a few ratio samples (1:10, 1:50 and 1:100) in a 20 µL amplification reaction containing 500 nM of each forward and reverse primer, 200 nM probe, 2× iTaq Universal Probes Supermix (Bio-Rad Laboratories Inc., Hercules, CA, USA), and water. After initial denaturation at 95 °C for 3 min, 40 cycles of amplification with a 15 s denaturation step at 95 °C and 1 min anneal and extension step at 60 °C were performed on a Bio-Rad CFX96 Real-Time PCR system (Bio-Rad Laboratories Inc.). After data capture, the amplification of unique products was verified in CFX Manager v3.1 (Bio-Rad Laboratories), and the primer pair with the lowest Cq value was selected (Table 1).

### 2.5. Real-Time PCR

The optimal concentration of the primers and probe and the optimal annealing temperature were determined with additional tests including varying the primer concentration from 125 nM to 875 nM and the probe concentration from 40 nM to 320 nM in each reaction and an annealing temperature gradient from 50 to 60 °C. The optimized real-time PCR reaction used iTaq Universal Probes Supermix (Bio-Rad Laboratories Inc., Hercules, CA, USA) with 500 nM of each primer and 160 nM probe, 2 µL DNA of varying concentration, and water to complete the dilution. In select assays, an 18S control probe and primer set were also used following the concentrations and reagents outlined in Barr et al. ([35]; Table 1). Real-time PCR was done on a Bio-Rad CFX96 Real-Time PCR system (Bio-Rad Laboratories Inc.). The following thermocycling protocol was used: 95 °C for 3 min followed by 40 cycles of 95 °C for 15 s, 1 min at 56 °C, and data capture. Data were visualized and analyzed in CFX Manager v3.1 (Bio-Rad Laboratories). Baseline threshold was set to “auto calculated” for initial testing. However, in a limited number of negative control samples (*H. zea* only and NTC), the diagnostic probe exhibited background amplification with an end relative fluorescence unit (RFU) of less than 50.00. Based on this, the baseline threshold setting for the diagnostic probe was changed to “user defined” and the cutoff value adjusted to 100.00 RFU for all runs.

### 2.6. Real-Time PCR Sensitivity Analyses

To determine the sensitivity of the assay, we ran six technical replicates of serial dilutions of purified *H. armigera* DNA with a range of concentrations from 40 ng/µL to 4 × 10^−6^ ng/µL. The Cq results were adjusted by a logarithmic regression at each DNA dilution for the diagnostic and control probes according to the model:

y_i_ = β_0_ + β_1_ log10 (X_i_) + e_i_,

where:

y_i_ = Cq observed value referring to the i-th dilution;
β_0_ = intercept;
β_1_ = slope;
X_i_ = the i-th dilution associated to the observed value y_i_;
e_i_ = residual associated to the y_i_ observation.

The analyses were carried out in R [36] using the nls2 package [37].

### 2.7. Real-Time PCR Comparative Analyses

The statistical significance within real-time PCR between the RFU amplification values of the purified and non-purified samples (use of bead purification factor levels; j) and the presence or absence of the 18S control probe (use of control factor levels; i) were evaluated by analysis of variance (ANOVA). Once the ANOVA assumptions were verified, the RFU value was modeled by the expression below:

y_ijk_ = µ + w_k_ + α_i_ + β_j_ + αβ_ij_ + e_ijk_,

where:

y_ijk_ = RFU observed value referring to the k-th bulk sample of combination of the i-th level of use of 18S control factor with the j-th level of use of bead purification factor;
µ = intercept;
w_k_ = effect of k-th bulk samples in the observed value y_ijk_;
α_i_ = effect of i-th level of the use of control factor in the observed value y_ijk_;
β_j_ = effect of j-th level of the use of bead purification factor in the observed value y_ijk_;
αβ_ij_ = effect of the interaction of the i-th level of the use of control factor as the j-th level of the use of bead purification factor;
e_ijk_ = residual associated with the y_ijk_ observation;

The analyses were carried out in R [36]. The means were compared by Tukey test using the agricolae package [38].

### 2.8. ddPCR

Primer and probe sets were also tested using ddPCR following the protocols outlined in Zink et al. [24] for EvaGreen reactions and Zink et al. [39] for probe-based reactions using the QX200 Droplet Digital PCR System (Bio-Rad Laboratories Inc.) The primer set was tested both with the probe and with EvaGreen intercalating dye (Table 1). The probe-based assay was carried out using 10 µL 2× ddPCR Supermix for Probes (no dUTP), 500 nM of each primer, 200 nM probe, 2 µL of DNA of varying concentration, and 5.6 µL water to complete the dilution of the master mix. After droplet generation, the following thermocycling protocol was used: 95 °C for 10 min followed by 40 cycles of 95 °C for 30 s, 56 °C for 30 s, and 72 °C for 1 min, concluding with 98 °C for 10 min and an infinite hold at 4 °C. During thermal cycling for ddPCR, the ramp rate between all steps was fixed at 2 °C/s.

The fully optimized EvaGreen assay was carried out using 10 µL EvaGreen Supermix (Bio-Rad Laboratories Inc.), 200 nM of each primer, 1–2 µL of DNA of varying concentration, and 7–8 µL of water to complete the dilution of the supermix. The following optimized thermocycling protocol was used for EvaGreen reactions: 95 °C for 5 min followed by 40 cycles of 95 °C for 30 s, 56 °C for 30 s, and 72 °C for 1 min, concluding with 4 °C for 5 min, 90 °C for 5 min, and an infinite hold at 4 °C. The ramp rate between steps was fixed at 2 °C/s.

After droplets were read, data were processed using ‘definetherain’ [40], and additional analyses were carried out in QuantaSoft v1.7.4.0917 (Bio-Rad Laboratories). The false positive rate (FPR) and Limit of Detection (LoD) for the primers was determined for the EvaGreen-based assay only. Forty-four replicates of bulk extractions containing *H. zea* specimens only were run, and the FPR and LoD were determined using look-up tables provided by Bio-Rad as modified from Armbruster and Pry [41].

## 3. Results

### 3.1. Real-Time PCR Assay Optimization

The ideal primer concentration for the real-time PCR assay was determined by conducting the assay with a range of primer concentrations. Increasing primer concentration generally resulted in an increase in Cq value. The end RFU values also increased with increasing primer concentration. A primer concentration of 500 nM was chosen for further testing because it optimized a low Cq and high end RFU (Supplementary Table S2). A gradient of probe concentrations from 40 to 320 nM was also tested while keeping primers constant at 500 nM. The Cq value decreased with increasing probe concentration through 240 nM at which point decreases in Cq were not substantially improved. The greatest increase in end RFU occurred between 120 nM and 160 nM. Therefore 160 nM was chosen as the optimal probe concentration (Supplementary Table S3).

The annealing temperatures for the primers and probe were optimized using a temperature gradient from 50–60 °C while keeping primer and probe concentrations at the empirically determined optima. The highest temperatures tested (between 56 °C and 60 °C) had the lowest Cq values, and 56.3 °C was ultimately chosen as the ideal melting temperature because it had the highest end RFU in the gradient (Supplementary Table S4). The annealing temperature was simplified to 56 °C for use in the remaining tests.

### 3.2. Real-Time PCR Sensitivity as Determined with High-Quality Template DNA from H. armigera

**Figure 1:**
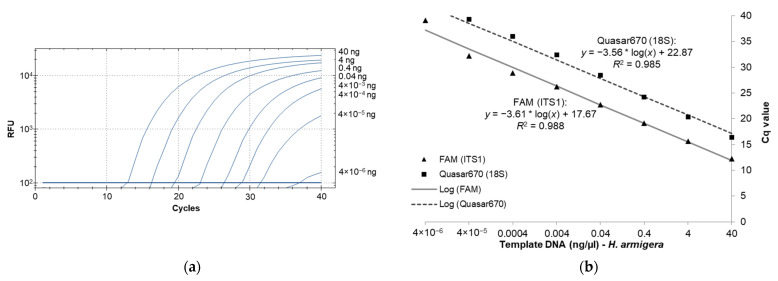
The diagnostic probe can detect the presence of *H. armigera* DNA across a wide dynamic range. (**a**) Amplification plot generated by serial dilution of *H. armigera* template DNA for the ITS1 diagnostic probes; (**b**) serial dilutions of template DNA for 6 *H. armigera* individuals with a standard. *: multiplication sign.

The standard curve for sensitivity was determined by plotting Cq values against a series of DNA template concentrations and showed an increase in Cq values as the template DNA concentrations decreased (Figure 1a). For the six samples used in generating the standard curve, the average Cq values ranged from 12.25 at 40 ng/µL to 39.10 at 4 × 10^−6^ ng/µL for the diagnostic probe and 16.37 at 40 ng/µL to 40 at 4 × 10^−6^ ng/µL for the 18S control probe. An R^2^ value of over 0.98 was obtained for both probes indicating a highly linear response across the range of DNA concentrations (Figure 1b). Based on sensitivity analysis, we experimentally determined 4 × 10^−5^ ng/µL as the minimum DNA concentration detectable by this assay, since the lower concentration (4 × 10^−6^ ng/µL) failed to produce a detectable signal in four out of the six tested samples.

### 3.3. Increased Salt Improves Squish Buffer Bulk Extractions

The DNA extraction was tested with three centrifugation speeds (2152, 8609, and 16,873× *g*), and the three following formulations of squish buffer: 10 mM Tris-HCl, 1 mM EDTA, and 25 mM NaCl ([30]); 10 mM Tris-HCl, 0.5 mM EDTA, and 12.5 mM NaCl [29]; and 10 mM Tris-HCl, 2 mM EDTA, and 50 mM NaCl. When the extractions were used for real-time PCR, we observed that samples with 1 leg of *H. armigera* and 20 legs of *H. zea* (1:20) exhibited a lower Cq value when the buffer with the highest salt concentration was used (Table 2).

More EDTA and NaCl was added to test samples using buffers with 2×, 5×, and 10× the concentration originally described in Gloor et al. [30]. When this set of extractions was used for real-time PCR, the buffer with 10× NaCl concentration exhibited an increased Cq value when paired with the 2152× *g* centrifugation speed, but the Cq value was unchanged at the higher centrifugation speed of 16,873× *g*. The lowest Cq value was attained with 5× EDTA and NaCl (an intermediate concentration) and using an intermediate centrifugation speed of 8609× *g* (Table 3).

### 3.4. Real-Time PCR Bulk Sample Results

With the optimal buffer formulation and centrifugation speed defined, the protocol was used to extract larger ratios of *H. zea* to *H. armigera*. Using the real-time PCR assay, we were able to successfully detect *H. armigera* DNA in the same PCR when it was co-extracted with *H. zea* DNA at ratios (*H. armigera*: *H. zea*) from 1:20 legs to 1:500 legs. The Cq values were > 0 for all ratios with *H. armigera* DNA with no Cq value for negative controls. The diagnostic probe returned Cq values of 26.14 for the ratio with the *most H. zea* legs, 1:500, and 17.15 for the ratio with the least *H. zea* legs, 1:20 (Table 4).

### 3.5. Bead Purification and Simplex Reactions Improve Sensitivity for Real-Time PCR Using Bulk Samples

**Figure 2:**
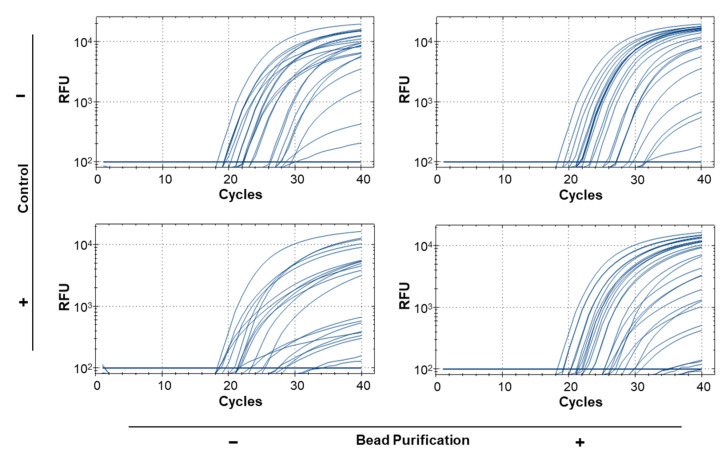
Real-time PCR reactions representing 59 samples of 1:50 (*H. armigera*: *H. zea*) legs. Samples were run 4 times: with and without bead purification and with and without the presence of the 18S control probe and primers.

To test the repeatability of real-time PCR results for bulk samples and find the sample size limit, we ran 59 replicates of 1:50 ratios using a single leg from H. armigera of varying quality and preservation to simulate old or degraded trap samples (Supplementary Table S1; Table 5). Of the 59 replicates, 12 returned false negative results (blank entries, Table 5) when using the modified squish buffer protocol for DNA extraction. Because the protocol does not include any purification steps, we hypothesized that this may be due the presence of PCR inhibitors. After the samples were purified using paramagnetic beads, the false negative rate was reduced to 2/59 (samples 56 and 57), and the end RFU for most samples increased, but no pattern was observed regarding Cq value (Table 5). This improvement was statistically significant when samples were compared before and after bead purification (*p* < 0.001; Table 6). Given previous work (e.g., [42,43,44]) showing that multiplex PCR can reduce amplification efficiency of some primers, we tested how inclusion or exclusion of the 18S control probe could affect identification of target DNA in bulk samples. Because there is competition for PCR reagents in the reaction, we ran the diagnostic and control probes independently for each sample. The unpurified samples had 6 false negative results while the bead purified samples had no false negatives for the diagnostic probe (Table 5). A significant difference (*p* < 0.001; Table 6) in end RFU values was found between reactions that included an 18S control probe and those that did not. However, no significant interaction was observed between bead purification and primer duplexing (*p* = 0.7472; Table 6). In addition to differences in identification rates, primer duplexing and bead purification produced significantly different end RFU values: the least square adjusted (LSA) mean of end RFU was 8215.243 (SE 213.068) for bead purified samples and 5104.481 (SE 211.826) for non-purified samples; the LSA mean was 8221.107 (SE 213.068) when the diagnostic probe was run in simplex and 5098.617 (SE 211.826) when run in duplex (Supplementary Figure S1). The difference in the shape of the curves can be visualized between the different methods with a steeper log phase evident for lower quality samples present after bead purification and lower Cq values for several samples when the diagnostic probe is run in simplex (Figure 2).

### 3.6. ddPCR Bulk Sample Results and Relative Performance of EvaGreen and Probe-Based Assays

**Figure 3:**
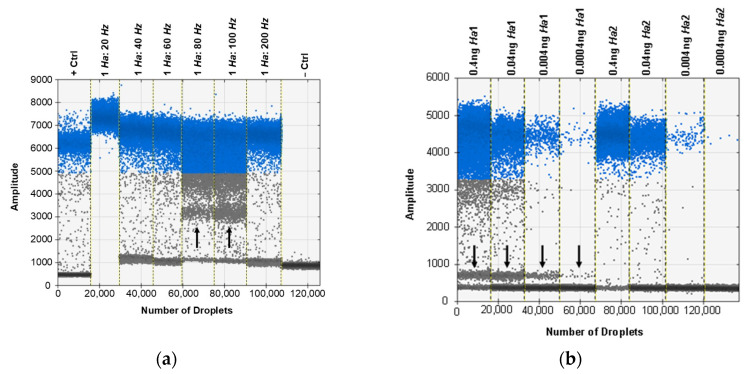
When the probe is used for ddPCR, an alternate cluster of droplets is evident for some samples. (**a**) The cluster (indicated by the black arrow) is prominent between the positive (blue) and negative (grey) droplets in lanes labeled 1 *Ha*: 80 Hz and 1 *Ha*: 100 Hz. (**b**) The cluster (indicated by the black arrow) is still evident after assay optimization and use of a touchdown thermocycling program. It is evident in the lanes labeled 0.4 ng *Ha*1 and 0.04 ng *Ha*1.

**Figure 4:**
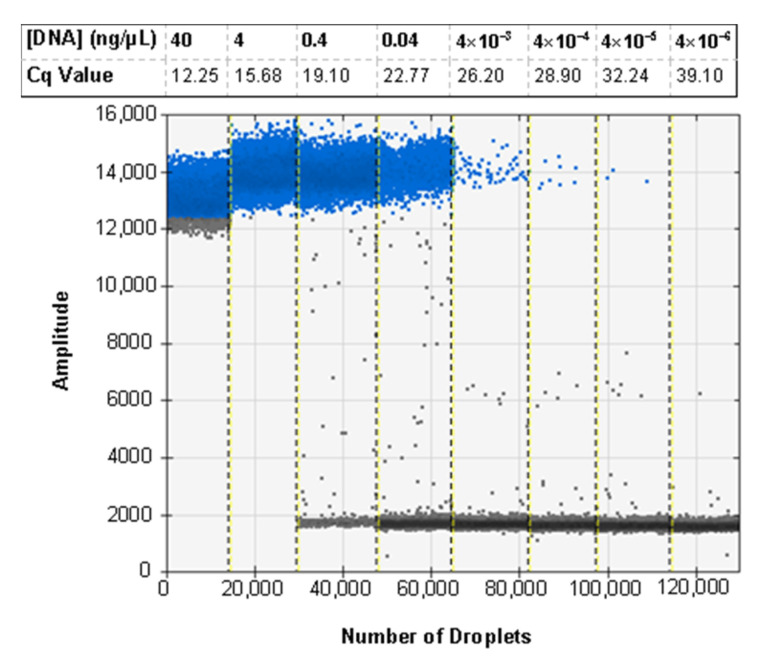
The ddPCR assay using EvaGreen is sensitive and able to detect *H. armigera* DNA to 4 × 10^−5^ ng/reaction. For comparison, the average Cq value for 6 technical replicates of the real-time PCR sensitivity assay is shown corresponding to the data presented in Figure 1. Positive droplets are shown in blue, and negative droplets are shown in grey apart from the lane marked 40 in which the grey droplets at the bottom of the positive cluster are likely falsely marked as negative due to the lack of a cluster of negative droplets in the well. This indicates that the assay is oversaturated with target DNA.

**Figure 5:**
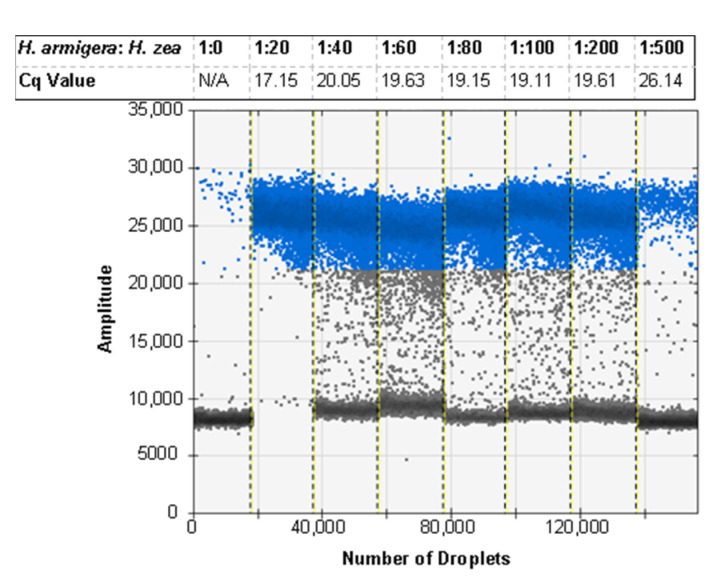
The ddPCR assay using EvaGreen is specific to *H. armigera* and can detect a single *H. armigera* leg in up to 500 *H. zea* legs. Cq values correspond to Table 4. Positive droplets are shown in blue, and negative droplets are shown in grey.

The ddPCR assay was first tested using the probe developed for the real-time PCR assay, but some specimens exhibited an unusual cluster of droplets of an intermediate amplitude between the positive and negative clusters. This was first seen when testing a selection of ratios of *H. zea* to *H. armigera* to demonstrate the sensitivity of the assay in bulk samples (Figure 3a). Through various optimization procedures to the ddPCR mixture and protocol, we were able to minimize but not eliminate the presence of the aberrant cluster (Figure 3b). While the result does not preclude using a probe as all samples returned the expected result, we proceeded to optimize the ddPCR assay for use with EvaGreen intercalating dye chemistry instead. The EvaGreen assay is as sensitive and specific as the probe-based assay and does not exhibit any abnormal amplification in the samples that were problematic with the probe. Both with and without the probe, the primers described here can be used to detect the lower limit of 4 × 10^−5^ ng/µL of target DNA in water. The EvaGreen assay can be used to detect *H. armigera* in the largest ratio tested, 1 *H. armigera*: 500 *H. zea* (Figure 4). The EvaGreen assay was not near failure at the 1:500 ratio and could likely detect a single *H. armigera* specimen in a far greater number of *H. zea*. The DNA was reduced to 1 µL per reaction to avoid saturating the droplet reader with positive droplets when testing the ratios with a high quantity of *H. armigera*. At 1:200 bulk samples using 1 µL of DNA per reaction, positive droplets still outnumbered negatives (Figure 5).

**Figure 6:**
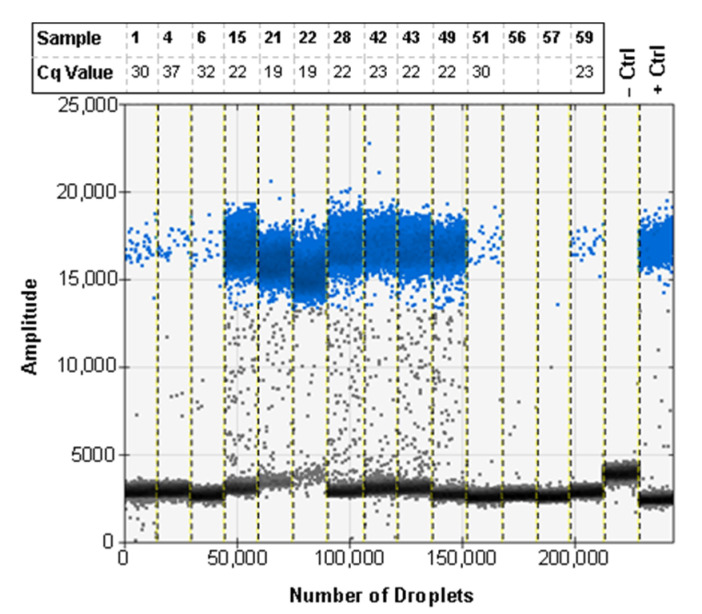
The ddPCR assay using EvaGreen can be used to detect *H. armigera* DNA in ratios of 1 *H. armigera* leg: 50 *H. zea* legs with low quality *H. armigera* specimens. The corresponding Cq value rounded to the nearest full number is shown for each of the samples. Sample names and Cq values correspond to Table 5. Blank entries indicate a false negative real-time PCR result. Positive droplets are shown in blue, and negative droplets are shown in grey.

Due to the nature of trap screening and the degradation that is associated with specimens exposed to field conditions, we tested the assay with a subset of the 1:50 ratio samples used for real-time PCR (Table 5). Of the samples run on ddPCR, 11 of 13 came back as positive for *H. armigera* (Figure 6). The two samples that were negative were also negative by real-time PCR when using the recommended method for screening.

### 3.7. ddPCR False Positive Rate

Due to the high levels of partitioning in ddPCR, determination of a false positive rate (FPR) for each assay is necessary. Because this assay is designed to be used with bulk samples, DNA extractions from *H. zea* were used to determine the FPR. The FPR for the EvaGreen assay is 0.36 droplets per well. The call threshold is 3 positive droplets, which indicates the fewest number of droplets that can reliably be called as a positive result. The limit of detection for this assay is 9 copies per well, which is the lowest number of copies of the gene of interest that can be present in a 20 µL PCR reaction that can reliably be detected as a true positive. Both thresholds are defined with a 99% confidence interval (Supplementary Figure S2).

## 4. Discussion

The use of ddPCR and real-time PCR has become mainstream for detection of target DNA from several different sample types [45]. Because each of these methods has advantages over the other and requires independent optimization, we developed, optimized, and compared assays for each platform. As part of the development and testing steps, the two methods were compared to help guide users as to which approach is most appropriate for their screening needs.

Given the reduced cost, ease of sample prep, wide availability, and the broad dynamic range associated with real-time PCR and because ddPCR is currently the only sensitive and specific method available for screening bulk samples of *Helicoverpa*, we focused on improving the sensitivity of detection for bulk samples by real-time PCR. Because ddPCR is generally more precise than real-time PCR when using complex and/or contaminated samples [46,47], our efforts were intended to enhance the real-time assay such that the sensitivity was closer to that realized with ddPCR. By using an increased salt concentration for the bulk extraction, secondary bead purification, and running the diagnostic assay in simplex, a significant increase in precision was observed over running the assay without these steps (Figure 2) as well as over previous attempts to develop a real-time PCR assay for use with bulk samples [24,29]. This is most evident in the 59 replicates of 1 *H. armigera*: 50 *H. zea* which were run under different control and purification conditions. While we observed that running the diagnostic and control probes separately resulted in no false negative results, we realized that this is an idealized situation that would not be applicable to routine procedures. In a real-world trap screening, running two separate assays to obtain diagnostic and control results is unrealistic as it uses twice the resources. Additionally, in a situation in which the diagnostic probe returned a negative result and the control probe returned a positive result, it would be impossible to know whether there was PCR occurring in the well with the diagnostic probe. Because of this, it would be more logical to split the trap sample into two or more small batches consisting of less than 50 moths each and run them with both probes simultaneously. This would ensure that the concentration of *H. armigera* DNA is relatively higher in any positive samples, increasing the probability of a correct diagnosis and allowing the control to work as intended. Our final recommendation is to bead purify all DNA extractions for use with this assay.

The most important aspect from these results is that the real-time PCR assay and the ddPCR assay returned positive results for the same samples. The two assays have the same sensitivity when used with purified DNA and can be used to detect 4 × 10^−5^ ng/µL of DNA per reaction at the lowest threshold (Figure 4). Both assays are also sensitive enough to detect a single *H. armigera* leg among 500 *H. zea* legs when fresh specimens are screened. The correlation between positive results for both assays shows that real-time PCR can be used successfully to screen for *H. armigera* in bulk samples using this assay, broadening the utility of this method for use by identifiers in labs that do not have access to ddPCR systems. Additionally, when the 59 1:50 ratio samples were run using real-time PCR, the two samples that return false negatives also did so when using ddPCR (Figure 6). This emphasizes the need to collect and properly store field samples promptly in order to preserve DNA of the highest quality possible.

The design of primers and a probe for this assay was optimized for the greatest number of fixed nucleotide differences between *H. zea* and *H. armigera* to ensure specificity in bulk samples. The largest difference between *H. zea* and *H. armigera* ITS1 sequences is a 30 bp deletion in *H. armigera* (5′ ACCACTATGCGCATGCATATATTGCATCGC). The forward primer was designed to span this deletion such that complete priming on *H. zea* by the forward primer was negated. The probe sequence immediately follows the forward primer and is designed to include two SNVs and an AA indel between the species. Like the probe, the reverse primer incorporates two SNVs and an AC indel separating the species.

In addition to high levels of lineage specific nucleotide sequence differences created from the rapid evolution in ITS sequences, increased sensitivity is also an advantageous feature of rDNA-based PCR assays due to the presence of high tandem copy numbers of rDNA in the genome [48]. Using a HiRise [49], assembled genome not available at the time of assay design [50] for *H. zea* x *H. armigera*, 54 *H. armigera* ITS1 sites (108 in diploid somatic cells), and 105 *H. zea* ITS1 sites (210 in diploid somatic cells) from distinct rDNA repeats were identified (Supplementary File S1). All 54 *H. armigera* ITS1 sites had 100% identity with each other and the primers and probe designed herein. In addition to the 54 distinct copies with exact matches for the primers and probe, four ITS1 copies were found that contained SNVs or indels (one copy with an indel in the forward priming site, one copy with one indel in the forward, probe, and reverse sequences, and two copies with one SNV each in the reverse sequence) in one or more of the priming sites. Because the birth and death cycle of rDNA is so rapid, the difference in rDNA copies is generally limited [51,52]. That said, given the large number of rDNA copies in a genome, it is common to find mutations between copies (ribotypes) at any given time especially in the non-functional ITS regions [53].

One of the most regularly cited problems associated with ddPCR assays is the production of aberrant droplets outside the expected range making analyses and detection calls more difficult [54]. Given this, our optimization steps were conducted to improve droplet separation and reduce rain and other out of range droplets. As with real-time PCR data, outputs (preferably sigmoidal curves in the case of real-time PCR and clearly separated droplets in the case of ddPCR) should always be visualized to ensure that positive calls are not being made from artifactual results [55,56]. Case in point, when the probe designed for real-time PCR was used in the ddPCR assay, we observed a tight and distinct cluster of positive droplets between the positive and negative droplet clusters (Figure 3a). We tried several optimization steps to eliminate or reduce the presence of these droplets including varying primer and probe concentration, as well as the annealing temperature, number of cycles, ramp rate, and implementing a touch-down-like thermocycling program. While the amplitude of the cluster could be reduced, it could not be eliminated entirely (Figure 3b).

The multi-peak distribution of droplets was found to be exclusive to the presence of the probe and may have been caused in part by incomplete or inefficient probe binding to ITS1 copies with a single nucleotide difference in the probe site [57]. When the *H. armigera* ITS1 copies are compared, the differential in ∆G (between perfect and next best matches) increases from −31.4 to −19.8 kcal/mol when the ITS1 copy with an indel in the probe binding site is considered. This suggests that increases in the frequency of minor ITS1 ribotypes may reduce the efficiency of probe binding and contribute to double banding. That said, despite the double banding in some samples, sufficient separation was noted between the double bands and the negatives such that positive calls could still be made after inspecting droplet amplitude. Furthermore, the samples that exhibited this droplet pattern were from colony samples of *H. armigera* and may not be representative of genotypes found widely in nature. No matter the reason for the multi-peak distribution found in some samples using the probe, the unusual pattern was eliminated in the EvaGreen ddPCR assay. While it is assumed that the off-target probe binding in the reaction is also present in the real-time PCR assay, the way in which the data are processed (a snapshot of total fluorescence in the reaction at each moment of data capture) make real-time assays less susceptible to these effects which are only evident due to the partitioning of DNA present in ddPCR. The advantages in specificity and sensitivity make rDNA loci superb diagnostic regions for species identification (even in the case of hybrids as shown in Supplementary File S1), but if possible, all ribotypes within a genome should be compared to improve reaction efficiency by avoiding intragenomic polymorphisms in priming sites.

**Figure 7:**
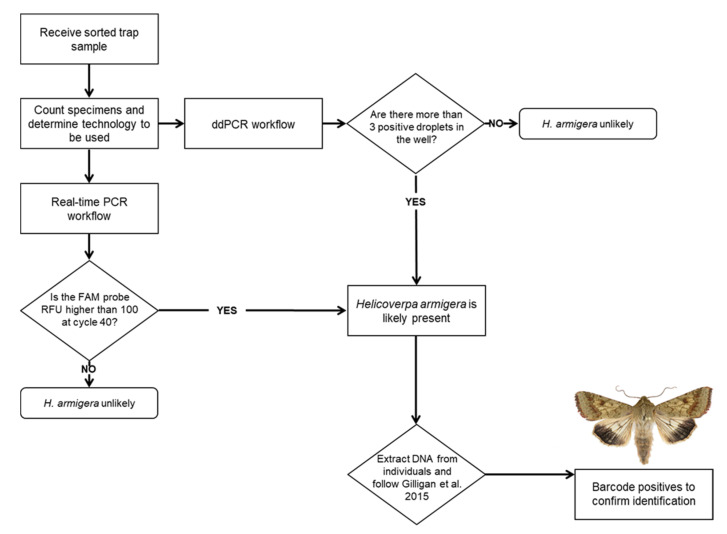
Flowchart describing assay choice and identification workflow.

The two assays described here were intended to improve the availability of rapid, sensitive detection methods for *H. armigera*. Since most identifiers rely on real-time PCR, barcoding, or genitalic dissection of individual specimens, any increased capacity for bulking samples with a widely available technology like real-time PCR is an important advancement for phytosanitary screening. Even our recommendation of bulking less than 50 specimens at a time for real-time will greatly improve throughput while maintaining a reasonable throughput for downstream identification. Currently, bulk samples are screened using the method described by Zink et al. [24], and if a bulk sample is determined to be positive for *H. armigera*, an individual leg is pulled from each specimen in the sample to extract DNA from it individually. The specimens are screened individually by real-time PCR following the guidelines from Gilligan et al. [31]. Positive samples are then COI barcoded, and/or the specimen is dissected for official identification. While the assay described by Zink et al. [24] can detect a single *H. armigera* in 999 *H. zea*, in practice much smaller samples are typically screened. Many traps catch fewer specimens with only a few hundred per trap collected during the peak of the season. Furthermore, if a sample containing hundreds of specimens is determined to be positive for *H. armigera*, individually extracting DNA from single legs of each of those specimens and running them each on real-time PCR becomes increasingly time consuming. In practice, it is more feasible to run fewer than 96 moths per trap sample so that any positive samples can be run on a single real-time PCR plate. The lower sample size also helps decrease the possibility of false negatives due to poor DNA quality. Similarly, the smaller sample size recommended here for this real-time PCR assay ensures the most efficient use of lab time and resources. In conclusion, we recommend dividing large trap catches into subsamples of 40 individuals to use the real-time PCR method (Figure 7).

## 5. Conclusions

Here, we show a comparison between real-time PCR and ddPCR for the purposes of detecting *H. armigera* in bulk trap samples. In the past, the accepted method for identifying *H. armigera* in complex samples containing many *H. zea* was to use ddPCR only. We sought to develop a real-time PCR assay that is similarly sensitive to a ddPCR assay such that either may be used by identifiers faced with screening a large number of samples, depending on the technology available to them. We show here that real-time PCR can be used to detect *H. armigera* in samples containing up to 50 *H. zea* with the same success rate as ddPCR. This was accomplished through the design of new primers and a new probe with high specificity to *H. armigera* as well as modifications to the currently accepted bulk DNA extraction technique that allow for higher quality input DNA through changes to the buffer formulation and the addition of a bead purification step. Not only is the real-time PCR assay now a viable option for screening large volume trap catches in a cost effective and timely manner, but the ddPCR assay presented here is superior to the currently used method by Zink et al. [24], as the primers are more specific and the FPR is much lower. The use of either the real-time PCR method or the ddPCR method will streamline the identification of this important pest and is a crucial tool for preventing establishment of the invasive species *H. armigera*.

## Figures and Tables

**Table 1:** Primers and probes used in this study.

Name	Description	Sequence	Tm (°C)	Source
Harm_18S_1944F	Diagnostic	5′-AACGTAAACAATAATCCACACACCA	55.1	This study
Harm_18S_2154R	Diagnostic	5′-CGCGGATTTTTGTGTTTTGTGT	56.5	This study
Harm_18S_1969P	Diagnostic	5′-6-FAM-CTAGAGGAC-ZEN-ACAGAGTCGAACG-IowaBlackFQ	56.4	This study
RT-18S-F2	Control	5′-ACCGCCCTAGTTCTAACCGTAAA	57.8	[35]
RT-18S-R2	Control	5′-CCGCCGAGCCATTGTAGTAA	57.3	[35]
RT-18S-P2	Control	5′-Quasar670-TGTCATCTAGCGATCCGCCGA-BHQ-2	60.3	[35]

**Table 2:** Real-time PCR results from squish buffer formulations and centrifugation speeds; all samples are ratios of 1 *H. armigera* leg: 20 *H. zea* legs.

*rcf* (xg)	NaCl Conc. (mM)	EDTA Conc. (mM)	Cq
2152	12.5	0.5	23.33
16,873	12.5	0.5	20.39
2152	25	1	21.96
16,873	25	1	19.16
2152	50	2	18.92
16,863	50	2	18.33

**Table 3:** Real-time PCR results from high salt formulations of squish buffer. Leg ratios are *H. armigera*: *H. zea*.

Leg Ratio	*rcf* (xg)	NaCl Conc. (mM)	EDTA Conc. (mM)	Cq
1:20	2152	50	2	19.00
1:20	16,873	50	2	18.29
1:20	2152	250	10	20.58
1:20	16,873	250	10	18.15
1:20	8609	100	5	17.16
1:60	8609	100	5	20.36

**Table 4:** Real-time PCR results from varying ratios of *H. armigera* legs: *H. zea* legs. The *rcf* (×g) was 8609, the NaCl concentration was 100 mM, and the EDTA concentration was 5 mM for all ratios. The *H. armigera* probe was FAM labeled.

Ratio	FAM Cq
1:20	17.15
1:40	20.05
1:60	19.63
1:80	19.15
1:100	19.11
1:200	19.61
1:500	26.14
1:0	18.44
0:1	
NTC	

**Table 5:** Real-time PCR results of ratios (1 *H. armigera* leg: 50 *H. zea* legs) with and without bead purification (BP) and presence and absence of 18S control primers and probe (Ctrl). Blank entries in “Cq” columns indicate false negative results.

	−Ctrl/−BP		−Ctrl/+BP		+Ctrl/−BP		+Ctrl/+BP	
Sample	Cq	RFU	Cq	RFU	Cq	RFU	Cq	RFU
1	31.65	198.94	30.13	2291.84		11.58	30.11	402.18
2		17.11	31.32	573.64		14.32	35.72	116.86
3	27.58	1208.05	28.10	4182.44	30.20	242.66	27.36	1547.18
4		10.99	34.51	216.99		14.23	36.53	107.33
5	25.07	3302.44	25.69	7287.27	25.36	980.38	26.04	1531.11
6		8.74	35.72	127.97		20.37	32.15	263.40
7	22.18	8860.18	22.43	12,029.25	21.59	1303.99	21.76	10,240.34
8	28.67	221.20	26.02	5089.04		22.56	25.97	2504.50
9	20.53	14,640.72	20.91	11,983.67	20.18	8987.33	21.09	4915.74
10	20.60	12,828.26	21.63	11,260.27	21.32	8552.34	21.87	7851.89
11	20.69	6733.72	21.12	14,552.49	20.78	5623.11	21.11	9787.14
12	26.21	2500.98	25.95	8619.49	25.51	1333.16	25.81	3735.53
13	26.69	7189.02	27.17	6005.49	25.14	591.95	27.02	5404.36
14	27.95	2879.51	26.02	9604.84	27.38	465.66	25.38	3628.56
15	19.83	12,057.90	21.83	12,285.58	20.30	9984.92	22.09	9215.15
16	20.50	14,147.07	21.46	12,672.84	20.44	7220.47	21.42	10,179.95
17	19.45	10,313.26	21.49	13,419.63	20.47	6204.70	21.32	10,309.40
18	19.73	10,536.28	21.11	13,353.52	20.62	9439.42	21.27	10,526.17
19	21.17	14,933.12	21.82	15,438.62	18.63	4943.59	21.12	12,605.58
20	19.28	14,301.02	21.78	15,255.25	19.73	8265.60	23.67	8443.85
21	19.60	7794.62	20.00	16,928.45	18.93	9460.50	19.16	13,813.90
22	19.18	9441.36	19.20	17,116.53	18.64	3999.75	19.09	14,047.80
23	21.21	10,290.38	21.31	14,504.58	21.43	11,550.59	21.94	11,009.13
24	29.23	229.14	27.10	6704.37	27.29	460.06	26.87	1772.06
25	25.03	3004.36	25.06	10,938.61	25.22	915.49	24.39	5906.28
26	21.25	9820.98	21.48	11,949.11	21.28	5401.31	21.24	11,309.96
27	20.37	11,952.59	19.72	15,220.42	20.37	9794.59	19.88	12,599.93
28	22.27	12,402.87	22.79	11,506.99	22.06	4630.17	22.07	9768.89
29	23.47	10,129.45	22.37	4684.52	23.39	3080.91	23.34	8869.53
30	21.65	9775.21	22.61	13,290.02	21.55	7357.58	22.21	6413.43
31	25.66	2216.63	22.72	6769.11	25.47	765.97	23.04	5099.04
32	20.23	11,324.96	20.26	16,288.94	19.95	7612.89	20.16	11,456.42
33	20.87	5143.82	19.55	16,991.30		23.07	19.43	11,137.10
34	20.25	13,967.67	20.69	15,999.46	20.90	11,097.20	20.39	12,280.23
35	22.06	11,644.17	21.87	15,842.31	21.53	3371.78	22.43	10,471.81
36	28.31	3564.74	28.53	2762.28	28.75	267.93	28.41	1097.53
37	22.12	6009.29	21.95	15,605.20	23.25	519.00	21.59	10,985.13
38	19.33	14,362.07	21.14	16,877.70	21.63	346.42	20.31	12,842.48
39	22.20	11,286.24	22.13	14,702.44	21.37	4716.22	21.38	10,587.83
40	23.65	8505.87	24.17	10,657.79	23.44	4196.02	23.69	8107.57
41	28.61	377.28	27.06	6554.71	33.05	140.50	26.21	2651.93
42	23.43	7592.74	23.35	12,718.12		17.83	22.75	9158.52
43	22.34	4626.78	22.43	11,485.15	28.37	252.15	22.42	9117.53
44	20.55	8772.13	20.12	17,240.06	21.34	1286.78	20.51	11,953.43
45	20.43	10,108.94	19.98	12,116.37	21.58	1636.07	20.40	10,375.38
46	25.23	2868.87	24.40	11,150.40	25.01	2595.12	24.80	3996.54
47	22.43	2316.19	22.52	7676.01	22.47	6662.04	23.15	6540.77
48		5.68	26.96	6957.75		10.08	27.34	508.59
49	22.12	5735.89	23.10	13,885.75	21.76	4549.79	22.07	10,361.06
50	30.12	185.46	27.07	4677.50	34.21	120.88	25.79	1623.45
51	29.38	1970.22	29.74	1305.81		37.49	29.96	977.40
52	26.19	7288.93	25.82	7727.24	25.63	2584.78	25.35	6046.17
53	26.95	4197.06	27.62	5171.64	27.50	298.00	27.43	864.95
54	24.32	9713.18	25.29	8753.72	24.69	2931.30	25.21	3978.88
55	22.44	11,339.61	23.09	3627.23	22.84	3692.81	23.28	5743.53
56		6.94	32.23	362.75		6.86		84.44
57		15.72	31.32	544.89		9.25		88.04
58	39.24	90.50 ^1^	31.40	582.00		8.51	34.41	141.23
59	22.31	9172.41	22.08	9900.71	22.07	5431.74	22.83	8343.40

^1^ Number reported is the average of the last 5 cycles recorded (as per machine default settings); threshold was only exceeded in the final cycle and is averaged with 4 below-threshold results.

**Table 6:** Analysis of variance table of end RFU values for samples with and without bead purification (BP) and presence and absence of 18S control primers and probe (Ctrl) as well as the interaction between the two factors (Ctrl × BP).

Variation Source	df	Sum Square	Mean Square	F	*p*
Bulk sample	58	3,952,285,666	68,142,856	13.018	<2 × 10^−16^ ***
Use of 18S control (Ctrl)	1	546,435,572	546,435,572	104.39	<2 × 10^−16^ ***
Use of bead purification (BP)	1	561,294,724	561,294,724	107.23	<2 × 10^−16^ ***
Ctrl × BP	1	545,735	545,735	0.1043	0.7472
Residuals	171	895,103,428	5,234,523		

*** Significant at *p* < 0.001 by the F test.

## Data Availability

All data generated and analyzed for this study are included in this published article and the associated Supplementary Information Files. Additional inquiries can be directed to the corresponding authors.

## Supplementary Materials

The following are available online at https://www.mdpi.com/article/10.3390/insects12100885/s1, Figure S1: Response to bead purification and internal control by Tukey test, Figure S2: ddPCR used for FPR and LoD, Table S1: sample IDs for 1:50 ratio DNA extractions, Table S2: Primer gradient to optimize real-time PCR, Table S3: Probe gradient to optimize real-time PCR, Table S4: Temperature gradient to optimize real-time PCR, File S1: ITS1 ribotypes of a *H. zea* x *H. armigera* hybrid.
