# Supplementary material for: Assay Optimization Can Equalize the Sensitivity of Real-Time PCR with ddPCR for Detection of Helicoverpa armigera (Lepidoptera: Noctuidae) in Bulk Samples
Source: Insects. 2021 Sep 29;12(10):885. doi: 10.3390/insects12100885 (PMC8538000; doi:10.3390/insects12100885)
Supplement: Supplementary file 1 [file insects-12-00885-s001.zip › Supp Fig S1.pdf]

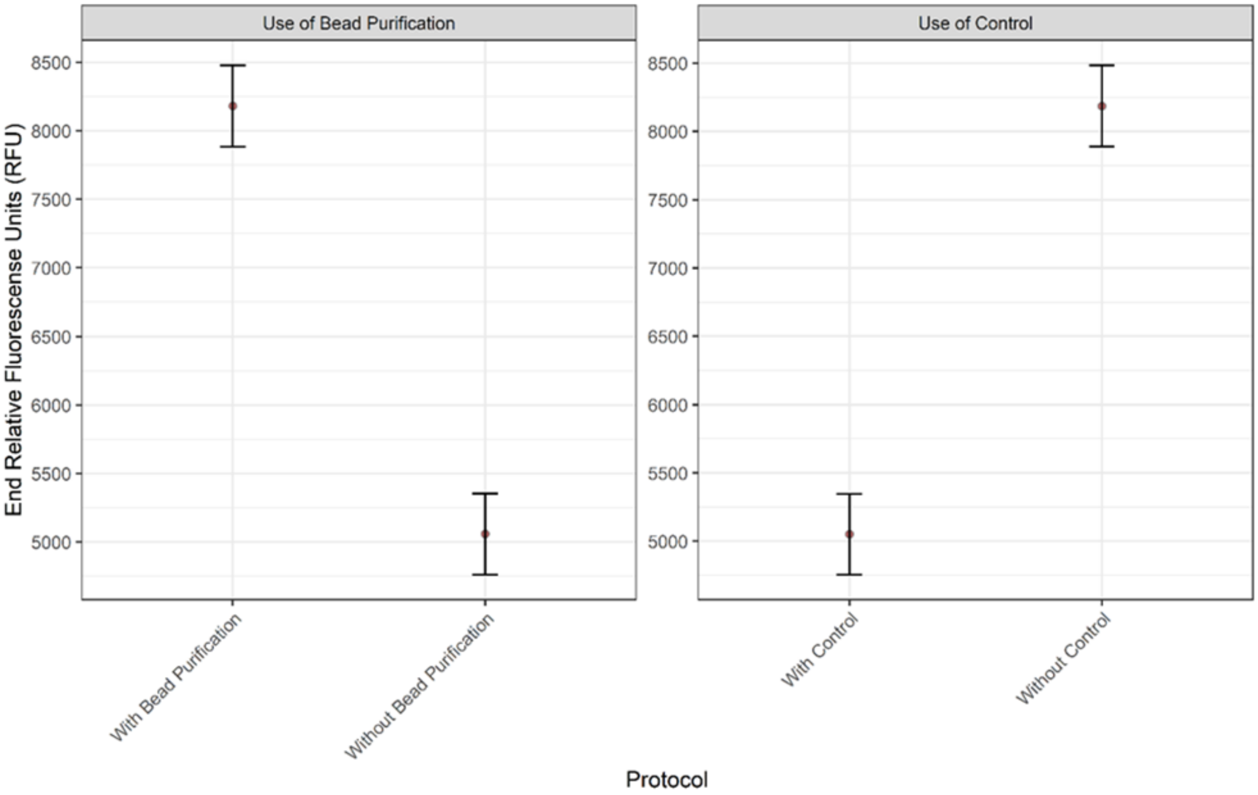

Supplementary Figure S1: Response in real-time PCR end RFU values across 59 bulk samples with and without bead purification and with and without the use of internal 18S control probes by Tukey test at 5% of significance. The bar represents the minimum significant difference of the test.
