# Supplementary material for: Assay Optimization Can Equalize the Sensitivity of Real-Time PCR with ddPCR for Detection of Helicoverpa armigera (Lepidoptera: Noctuidae) in Bulk Samples
Source: Insects. 2021 Sep 29;12(10):885. doi: 10.3390/insects12100885 (PMC8538000; doi:10.3390/insects12100885)
Supplement: Supplementary file 1 [file insects-12-00885-s001.zip › Supp Fig S2.pdf]

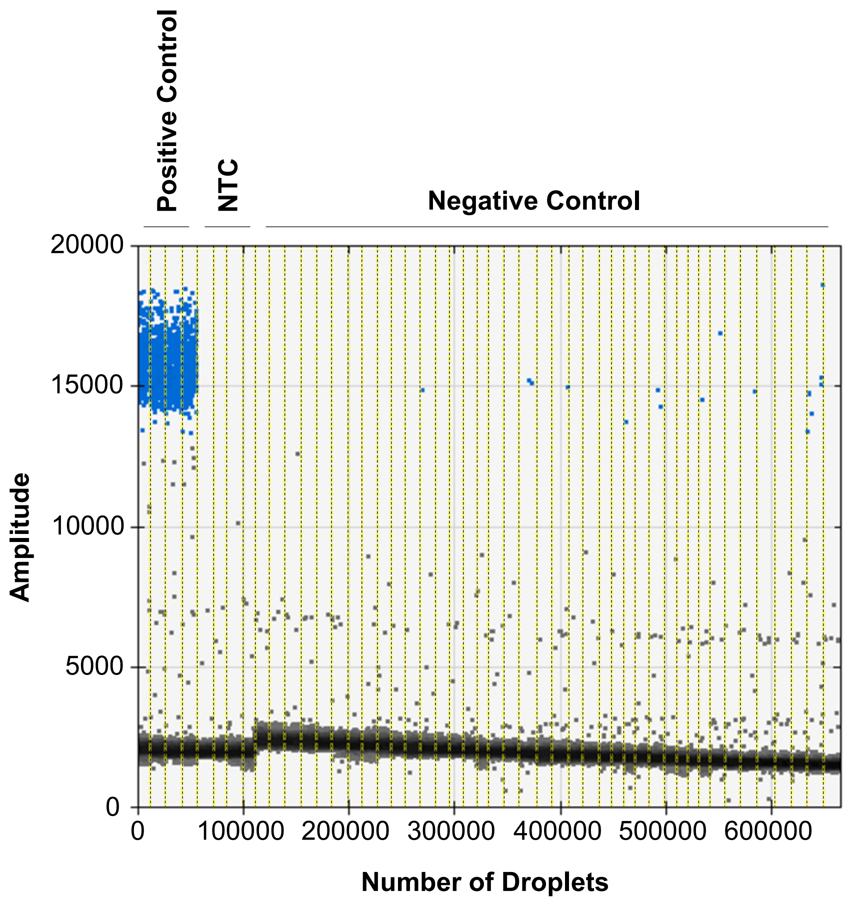

Supplementary Figure S2: False positive rate and limit of detection were determined for the ddPCR assay using EvaGreen by running DNA extractions from 50 *H. zea* legs. Positive droplets are shown in blue, negative droplets are shown in grey.
