# Supplementary material for: Assay Optimization Can Equalize the Sensitivity of Real-Time PCR with ddPCR for Detection of Helicoverpa armigera (Lepidoptera: Noctuidae) in Bulk Samples
Source: Insects. 2021 Sep 29;12(10):885. doi: 10.3390/insects12100885 (PMC8538000; doi:10.3390/insects12100885)
Supplement: Supplementary file 1 [file insects-12-00885-s001.zip › Supplementary Table S1.pdf]

Supplementary Table S1: 1:50 ratio replicate IDs

Sample	*H. armigera* origin	Year	*H. zea* origin	Year
1	Queensland, Aus	2015	Mayo, FL, USA	2019
2	Queensland, Aus	2015	Mayo, FL, USA	2019
3	Queensland, Aus	2015	Mayo, FL, USA	2019
4	Queensland, Aus	2015	Mayo, FL, USA	2019
5	Queensland, Aus	2015	Mayo, FL, USA	2019
6	Queensland, Aus	2015	Mayo, FL, USA	2019
7	Queensland, Aus	2015	Mayo, FL, USA	2019
8	Queensland, Aus	2015	Mayo, FL, USA	2019
9	Puerto Rico lab colony	2016	Mayo, FL, USA	2019
10	Puerto Rico lab colony	2016	Mayo, FL, USA	2019
11	Puerto Rico lab colony	2016	Mayo, FL, USA	2019
12	Puerto Rico lab colony	2016	Mayo, FL, USA	2019
13	Puerto Rico lab colony	2016	Mayo, FL, USA	2019
14	Puerto Rico lab colony	2016	Mayo, FL, USA	2019
15	Franschhoek, S.A.	2017	Mayo, FL, USA	2019
16	Franschhoek, S.A.	2017	Mayo, FL, USA	2019
17	Franschhoek, S.A.	2017	Mayo, FL, USA	2019
18	Franschhoek, S.A.	2017	Mayo, FL, USA	2019
19	Franschhoek, S.A.	2017	Mayo, FL, USA	2019
20	Franschhoek, S.A.	2017	Mayo, FL, USA	2019
21	Franschhoek, S.A.	2017	Mayo, FL, USA	2019
22	Franschhoek, S.A.	2017	Mayo, FL, USA	2019
23	Franschhoek, S.A.	2017	Mayo, FL, USA	2019
24	Mbombela, S.A.	2017	Mayo, FL, USA	2019
25	Mbombela, S.A.	2017	Mayo, FL, USA	2019
26	Paarl, S.A.	2017	Mayo, FL, USA	2019
27	Paarl, S.A.	2017	Mayo, FL, USA	2019
28	Paarl, S.A.	2017	Mayo, FL, USA	2019
29	Paarl, S.A.	2017	Mayo, FL, USA	2019
30	Philippi, S.A.	2017	Mayo, FL, USA	2019
31	Philippi, S.A.	2017	Mayo, FL, USA	2019
32	Philippi, S.A.	2017	Mayo, FL, USA	2019
33	Philippi, S.A.	2017	Mayo, FL, USA	2019
34	Philippi, S.A.	2017	Mayo, FL, USA	2019
35	Philippi, S.A.	2017	Mayo, FL, USA	2019
36	Philippi, S.A.	2017	Mayo, FL, USA	2019
37	Philippi, S.A.	2017	Mayo, FL, USA	2019
38	Philippi, S.A.	2017	Mayo, FL, USA	2019
39	Otis lab colony	2017	Mayo, FL, USA	2019
40	Otis lab colony	2017	Mayo, FL, USA	2019
41	Otis lab colony	2017	Mayo, FL, USA	2019
42	Otis lab colony	2017	Mayo, FL, USA	2019
43	Zululand, S.A.	2017	Mayo, FL, USA	2019
44	Zululand, S.A.	2017	Mayo, FL, USA	2019
45	Paarl, S.A.	2019	Mayo, FL, USA	2019
46	Paarl, S.A.	2019	Mayo, FL, USA	2019

47	Paarl, S.A.	2019	Mayo, FL, USA	2019
48	Paarl, S.A.	2019	Mayo, FL, USA	2019
49	Paarl, S.A.	2019	Mayo, FL, USA	2019
50	Paarl, S.A.	2019	Mayo, FL, USA	2019
51	Queensland, Aus	2019	Mayo, FL, USA	2019
52	Queensland, Aus	2019	Mayo, FL, USA	2019
53	Queensland, Aus	2019	Mayo, FL, USA	2019
54	Simondium, S.A.	2019	Mayo, FL, USA	2019
55	Simondium, S.A.	2019	Mayo, FL, USA	2019
56	Simondium, S.A.	2019	Mayo, FL, USA	2019
57	Simondium, S.A.	2019	Mayo, FL, USA	2019
58	Wellington, S.A.	2019	Mayo, FL, USA	2019
59	Wellington, S.A.	2019	Mayo, FL, USA	2019
