# Supplementary material for: Assay Optimization Can Equalize the Sensitivity of Real-Time PCR with ddPCR for Detection of Helicoverpa armigera (Lepidoptera: Noctuidae) in Bulk Samples
Source: Insects. 2021 Sep 29;12(10):885. doi: 10.3390/insects12100885 (PMC8538000; doi:10.3390/insects12100885)
Supplement: Supplementary file 1 [file insects-12-00885-s001.zip › Supplementary Table S2.pdf]

Supplementary Table S2: real-time PCR results from primer concentration gradient; probe at 200nM

**Primer conc. (nM)**	**Cq**	**End RFU**
125	18.72	11752.66
250	18.70	22008.61
375	18.94	21939.63
500	18.96	24868.50
625	19.06	23687.81
750	18.89	25870.61
875	19.11	24776.83
0		3.32
