# Supplementary material for: Assay Optimization Can Equalize the Sensitivity of Real-Time PCR with ddPCR for Detection of Helicoverpa armigera (Lepidoptera: Noctuidae) in Bulk Samples
Source: Insects. 2021 Sep 29;12(10):885. doi: 10.3390/insects12100885 (PMC8538000; doi:10.3390/insects12100885)
Supplement: Supplementary file 1 [file insects-12-00885-s001.zip › Supplementary Table S3.pdf]

Supplementary Table S3: real-time PCR results from probe concentration; primers at 500nM each

Probe conc. (nM)	Cq	End RFU
40	23.12	7809.71
80	22.02	14038.24
120	21.59	16992.24
160	21.3	21147.71
200	21.17	22859.44
240	21.13	24143.71
280	21.13	24657.33
320	21.13	24853.07
0		4.96
