# Supplementary material for: Assay Optimization Can Equalize the Sensitivity of Real-Time PCR with ddPCR for Detection of Helicoverpa armigera (Lepidoptera: Noctuidae) in Bulk Samples
Source: Insects. 2021 Sep 29;12(10):885. doi: 10.3390/insects12100885 (PMC8538000; doi:10.3390/insects12100885)
Supplement: Supplementary file 1 [file insects-12-00885-s001.zip › Supplementary Table S4.pdf]

Supplementary Table S4: real-time PCR results from temperature gradient with primers at 500nM and probe at 160nM

Annealing temp. (°C)	Cq	End RFU
60	21.96	18195.06
59.4	21.92	19047.54
58.3	21.94	19313.38
56.3	21.96	20336.74
53.9	22.03	20248.93
52	22.08	18829.02
50.7	22.07	18160.84
50	22.19	16570.87
